# Anti-MAG neuropathy: historical aspects, clinical-pathological correlations, and considerations for future therapeutical trials

**DOI:** 10.1055/s-0043-1777728

**Published:** 2024-02-07

**Authors:** Norman Latov, Thomas H. Brannagan, Howard W. Sander, Francisco de Assis Aquino Gondim

**Affiliations:** 1 Weil Medical College of Cornell University, Peripheral Neuropathy Center, New York, New York, United States.; 2 Columbia University, Vagelos College of Physicians and Surgeons, Peripheral Neuropathy Center, Department of Neurology, New York, New York, United States.; 3 New York University, Department of Neurology, New York, New York, United States.; 4 Universidade Federal do Ceará, Departamento de Medicina Clínica, Serviço de Neurologia, Fortaleza CE, Brazil.

**Keywords:** Demyelinating Diseases, Anti-MAG, Neuropathy, Doenças Desmielinizantes, Anti-MAG, Neuropatia

## Abstract

**Background** Patients with anti-MAG neuropathy present with distal demyelinating polyneuropathy, IgM monoclonal gammopathy, and elevated titers of anti-MAG antibodies.

**Objective** This paper reviews what is known about the clinical presentation, course, pathophysiology, and treatment of anti-MAG neuropathy, with considerations for the design of therapeutic trials.

**Methods** A literature review of the medical and scientific literature related to anti-MAG neuropathy, and the design of therapeutic clinical trials in peripheral neuropathy.

**Results** Anti-MAG neuropathy can remain indolent for many years but then enter a progressive phase. Highly elevated antibody titers are diagnostic, but intermediate titers can also occur in chronic inflammatory demyelinating polyneuropathy (CIDP). The peripheral nerves can become inexcitable, thereby masking the demyelinating abnormalities. There is good evidence that the anti-MAG antibodies cause neuropathy. Reduction of the autoantibody concentration by agents that target B-cells was reported to result in clinical improvement in case series and uncontrolled trials, but not in controlled clinical trials, probably due to inadequate trial design.

**Conclusion** We propose that therapeutic trials for anti-MAG neuropathy include patients with the typical presentation, some degree of weakness, highly elevated anti-MAG antibody titers, and at least one nerve exhibiting demyelinating range abnormalities. Treatment with one or a combination of anti-B-cell agents would aim at reducing the autoantibody concentration by at least 60%. A trial duration of 2 years may be required to show efficacy. The neuropathy impairment score of the lower extremities (NIS-LL) plus the Lower Limb Function (LLF) score would be a suitable primary outcome measure.

## Resumo

**Antecedentes** Pacientes com neuropatia anti-MAG apresentam polineuropatia desmielinizante distal, gamopatia monoclonal IgM e títulos elevados de anticorpos anti-MAG.

**Objetivo** Este artigo revisa o que se sabe sobre a apresentação clínica, curso, fisiopatologia e tratamento da neuropatia anti-MAG, com considerações para o desenho de ensaios terapêuticos.

**Métodos** Revisão bibliográfica da literatura médica e científica relacionada à neuropatia anti-MAG e desenho de ensaios clínicos terapêuticos em neuropatia periférica.

**Resultados** A neuropatia anti-MAG pode permanecer indolente durante muitos anos, mas depois entra numa fase progressiva. Títulos de anticorpos altamente elevados são diagnósticos, mas títulos intermediários também podem ocorrer na polineuropatia desmielinizante inflamatória crônica (CIDP). Os nervos periféricos podem tornar-se inexcitáveis, mascarando, assim, as anomalias desmielinizantes. Há boas evidências de que os anticorpos anti-MAG causam a neuropatia. Foi relatado que a redução da concentração de autoanticorpos por agentes direcionados às células B resultou em melhora clínica em séries de casos e ensaios não controlados, mas não em ensaios clínicos controlados, provavelmente devido ao desenho inadequado dos ensaios.

**Conclusão** Propomos que os ensaios terapêuticos para neuropatia anti-MAG incluam pacientes com apresentação típica, algum grau de fraqueza, títulos de anticorpos anti-MAG altamente elevados e pelo menos um nervo exibindo anormalidades na faixa desmielinizante. O tratamento com um ou uma combinação de agentes anticélulas B teria como objetivo reduzir a concentração de autoanticorpos em pelo menos 60%. Pode ser necessária uma duração de ensaio de 2 anos para demonstrar eficácia. A pontuação de comprometimento da neuropatia das extremidades inferiores (NIS-LL) mais a pontuação da função dos membros inferiores (LLF) seria uma medida de resultado primário adequada.

## INTRODUCTION

### Historical aspects, epidemiology, and pathophysiology

The association of IgM monoclonal gammopathy with demyelinating neuropathy and anti-MAG antibodies was first reported by Latov and colleagues in 1980, 1 2 3 and subsequently confirmed by other investigators. 4 5 6

The prevalence of anti-MAG neuropathy is estimated to be between 0.45 and 1.2 per 100,000, with the lower number based on a survey in Southern England, 7 and the latter from epidemiologic studies reporting a prevalence of IgM monoclonal gammopathy of 51 per 100,000, 8 with 5.4% of Waldenström's patients experiencing neuropathy, 9 and approximately 50% of IgM proteins of patients with neuropathy exhibiting anti-MAG antibody activity. 10 In a large study of 202 patients, the male-to-female ratio was 1.9 to 1, with a mean age of symptom onset of 62.6 years (range 25-91). 11

There is considerable evidence that the neuropathy is caused by the monoclonal IgM anti-MAG antibodies. 12 13 The target antigen, MAG, is a component of peripheral nerve myelin. Anti-MAG antibodies immunostain myelin. 14 Sural nerve biopsies from affected patients show demyelination, widened myelin lamellae or unraveling of compact myelin at the minor dense line, and deposits of the monoclonal IgM and complement on the myelin sheaths, and in particular at the paranodes, Schmidt Lanterman clefts, and widened myelin lamellae, consistent with the known distribution of MAG. 15 16 17 18 Intraneural injection of anti-MAG antibodies induces demyelination in experimental nerves. 19 20 21 Passive transfer of anti-MAG IgM into experimental chicks induced demyelination, widening of the myelin lamellae, with deposits of IgM concentrated at the nodes of Ranvier, and Schmidt-Lanterman incisures, similar to that seen in affected human nerves. 22 The demyelination in the intraneural injection studies was complement-dependent, but the role of complement in the passive transfer chick studies was not investigated. It is likely that anti-MAG antibodies exert their effects via both complement-dependent and independent mechanisms. Other factors, such as permeability of the blood-nerve barrier, cytokines, macrophage activation, or complement regulatory molecules such as CD59 may also play a role in the development of neuropathy. 23 24 25 26 Such factors might explain the acute exacerbations that are sometimes seen following rituximab treatments, in the absence of an increase in antibody concentrations, presumably due to cytokine release, 27 or the transient improvement that can occur following therapy with IVIG. 28

### Clinical presentation and course

Patients with MAG neuropathy typically present with a chronic and slowly progressive distal demyelinating neuropathy, beginning with distal paresthesias or large fiber sensory loss and ataxia that spreads proximally with weakness and sensory loss. In a large study of 202 patients, 83% presented with the typical phenotype, but 17% had atypical presentations that included acute or chronic sensorimotor polyradiculopathies, and asymmetric or multifocal neuropathies, and 30% had a tremor. 11 Magy and colleagues 29 reported that 28% of patients presented with distal paresthesias only, whereas 53% had some level of weakness. Other studies also reported weakness in approximately half of the patients. 30 31 The tremor in anti-MAG neuropathy appears to be neurogenic, 32 is often unrelated to the severity of the underlying neuropathy, and often non-responsive to treatment, 33 as has also been described in CIDP. 34

Atypical presentations can result from other properties of the monoclonal gammopathies. Amyloid deposition of the monoclonal light chains has been associated with painful, autonomic, 35 asymmetric, 36 or cranial neuropathies. 37 Cryoglobulin deposition of the M-protein can be associated with multifocal vasculitic neuropathy. 38 39 Infiltration by B-cell lymphoma cells can be associated with mononeuritis multiplex. 40 41

The neuropathy can remain stable or be slowly progressive for many years, but then enter a more rapidly progressive course that can lead to disability, resulting from cumulative nerve damage with secondary axonal degeneration. 42 43 44 45 The disability rate is 16% at 5 years, 24% after 10 years, and 50% after 15 years. 46 There is no correlation between antibody titers and the severity of the neuropathy, although increasing antibody titers are probably indicative of expansion of the monoclonal B-cell population.

In the study by Svahn and colleagues, 11 approximately 68% had monoclonal gammopathy of unknown significance (MGUS), 29% had Waldenström's macroglobulinemia, and 3% had chronic lymphocytic leukemia or B-CLL. Castellani and colleagues reported that 66.7% of 75 patients tested had a mutation in MYD88, more often in macroglobulinemia 47 48 making them susceptible to treatment with Bruton tyrosine kinase inhibitors.

### Serological studies

Anti-MAG antibodies are routinely tested for by Enzyme-Linked Immunosorbent Assays (ELISA). 12 The two most commonly used assays are 1) The Buhlmann assay (Buhlmann Laboratories AG, Switzerland), a commercially available kit that compares the binding of the tested sera to a standard curve, and 2) a dilutional endpoint assay the results of which are given as the highest dilution at which binding above background is observed. In general, highly elevated anti-MAG antibody titers are more likely to be specific or diagnostic than mild or intermediate elevations that are higher than normal, but not necessarily pathogenic. Normal titers are ≤800 in the dilutional endpoint assay and ≤1000 in the Buhlmann assay, but titers of ≥1:25,600 in the dilutional endpoint assay 49 or ≥7,500 to 10,000 in the Buhlmann assay, are more likely to be associated with typical MAG neuropathy. 29 50 In patients with intermediate titers, the distinction from chronic inflammatory demyelinating polyneuropathy (CIDP) can be problematic given the overlapping clinical and electrophysiological features, and lack of a diagnostic test for CIDP, 51 although the presence of IgM myelin deposits on sural nerve biopsy would be diagnostic of anti-MAG neuropathy.

For B-cell-depleting therapies, the change in serum IgM concentration can be used to monitor the antibody response as it generally parallels the change in the concentration of the anti-MAG IgM. The Buhlmann assay can also be used to follow antibody titers, but the sera would have to be tested on the same ELISA plate at the same time for comparison, due to potential differences between kits, test conditions, or standards. The dilutional endpoint assay is less sensitive to change, as it is exponential rather than linear, so that a change of at least 50% would be required to detect a change in titer. 12

### Electrodiagnostic studies

Electrophysiologic studies in anti-MAG neuropathy typically show demyelinating abnormalities, consistent with the known pathophysiology. Nerve conduction velocities can be severely slowed early on, including in patients with relatively mild clinical deficits, likely resulting from chronic subclinical disease activity. Disability is more closely correlated with a reduction of CMAP amplitudes indicative of axonal loss. 43 The lower limb motor and sensory potentials show a marked trend to become unexcitable over time, 45 so that the studies may not meet EFNS/PNS demyelinating criteria, with the percentage of patients meeting the criteria reported as ranging from 20.9 to 91%. 29 30 42 45 In the study reporting 20.9% however, at least 1 nerve in all patients showed demyelinating range abnormalities. 42 Distal accentuation of conduction velocity slowing has also been described in a variable number of patients. 29 30 42 49 52 53 54 55 56

### Therapy

Therapy of anti-MAG neuropathy is directed at reducing the concentration of the monoclonal IgM autoantibody concentrations using chemotherapeutic agents, such as chlorambucil, cyclophosphamide, or fludarabine, or with rituximab, a therapeutic monoclonal antibody that targets CD20 on B-cells, with the latter being the preferred therapy, due to the need for chronic treatment and cumulative toxicities of the chemotherapeutic agents. 28 More recently, treatment with the anti-Bruton's tyrosine kinase (BTK) agents ibrutinib 57 or tirabrutinib, 58 or with venetoclax, an anti-B-cell lymphoma-2 (BLCl2) agent that promotes apoptosis, 59 have also been reported to be of benefit. A retrospective analysis of 50, mostly uncontrolled studies with 410 patients found that a relative reduction in the serum IgM anti-MAG antibody level is associated with a clinical response. 60 IgM MGUS and Waldenström's associated anti-MAG neuropathies displayed similar responses. 61

A series of 25 patients treated with a combination of rituximab/cyclophosphamide/prednisolone every 21 days for 6 cycles showed improvement in the overall neurological limitations scale (ONLS), a derivation of the INCAT scale which improved from a median of 3 to 2 at year 1 and 2 to 1 at year 2. Among the patients with weakness, there was an improvement of 69% in the MRS sum scale. The ISS improved by 50%. Electrophysiologic studies showed improvement in the mean distal motor latency score and the sensory nerve action potential (SNAP) amplitude sum score. 62

Two placebo-controlled studies of IVIG in IgM-associated neuropathy did not meet their primary endpoint. In a placebo-controlled crossover study, 11 patients were randomized to 2gm/kg IVIG or placebo monthly for three months followed by the other treatment. 63 No significant differences were seen in the endpoints other than improved strength in 2 of 11 patients and an improved sensory score in one patient. In a double-blind crossover study, 22 patients (50% of whom had anti-MAG antibodies) were randomized to 2gm/kg IVIG or placebo followed by the other treatment. There was no difference between the two groups in the primary outcome of INCAT disability score at two weeks although there was improvement at four weeks (secondary outcome) in the IVIG group. 64 Two small controlled clinical trials of rituximab in anti-MAG neuropathy failed to show a therapeutic benefit, 65 66 although a meta-analysis concluded that there is low-quality evidence of a benefit from this treatment. 28 The Dalakas study 65 included 26 patients, with the treatment group given a single course of rituximab and followed for 8 months, using the INCAT score as the primary outcome measure. This study showed a statistically significant change (favoring the rituximab group) in the 10-meter walk test, which was a secondary outcome. The Leger study 66 included 54 patients, with the treatment group given a single course of rituximab and followed for 12 months, using the INCAT Sensory Score (ISS) as the primary outcome measure. Possible reasons for the studies' failures include size, patient selection, regimen, duration, or choice of outcome measures. 67 68

## CONSIDERATIONS FOR FUTURE CLINICAL TRIALS

### Patient selection

Given the potential overlap with CIDP, it would be prudent to include patients with the typical phenotype, with at least 1 nerve exhibiting demyelinating range abnormalities, with an IgM monoclonal gammopathy (MGUS or Waldenström's macroglobulinemia), and highly elevated anti-MAG antibodies of ≥7,500 using the Buhlmann assay or ≥1:25,600 in the dilutional assay, as previously discussed.

Due to the indolent course of the disease, more likely progression in patients with weakness, and relative insensitivity of outcome measures assessing mild gait instability, it would be preferable to include patients with some degree of weakness, even if it is only at the toes, or difficulty rising on the heels or toes. Such patients are more likely to be in a progressive phase, and changes in strength are more readily detected and quantified than changes in sensation or gait alone.

A longer trial duration of 2 years with rituximab or another anti-CD20 agent, 69 and continued treatment would increase the likelihood of detecting a difference between treated and non-treated patients. Continued treatment may be required to sufficiently lower the antibody concentration or prevent a rebound, and subjects in the placebo group are more likely to worsen and less likely to exhibit a placebo response over the longer time period. In the Dalakas study, as an example, a single course of treatment with rituximab reduced IgM levels at 8 months by only 34%. 65 Addition of BKT inhibitors in those with the MYD88 mutation or with venetoclax which targets BCL2 could also be considered in cases where the IgM levels are insufficiently responsive to therapeutic anti-CD20 antibodies at the 6-month time period.

With regards to the primary outcome measure, the neuropathy impairment score (NIS) of the lower extremities (NIS-LL) plus Lower Limb Function (LLF) 70 is likely to be more suitable for anti-MAG neuropathy trials than previously used measures. The NIS-LL measures both manual muscle strength and sensory functions including at the toes and ankles, and the LLF also measures functional strength at the ankles, whereas the INCAT score of the lower extremities only assesses gait. The NIS score has been used in clinical trials of hereditary amyloidosis which also presents with distal weakness and sensory loss, 71 whereas the INCAT score has mostly been used in trials of CIDP which typically presents with both proximal and distal weakness. 72 The MRC score can also be used for grading muscle strength but is best suited for patients with more severe weakness. 73 Walking speed, or the 10-meter walk test, which demonstrated a significant improvement in the treatment vs control groups in the Dalakas but not Leger study, would also be of interest, especially with the inclusion of more sensitive gait parameters that can be electronically measured. 27 Given that many of the patients in both arms of the trial may not show a change given the indolent nature of the disease, the use of two independent measures such as the number improving or worsening, or a combination of outcome measures might show a greater treatment effect. 74 These issues could be best addressed in collaborative multicenter studies such as the ongoing IMAGiNe study. 75
